# Unveiling the Immunomodulatory Potential of Finger Millet Components: A High-Resolution Mass Spectrometry Analysis

**DOI:** 10.7759/cureus.72547

**Published:** 2024-10-28

**Authors:** Saroj Adittya Rajesh, K. Ramachandra Reddy

**Affiliations:** 1 Department of Integrative and Complementary Medicine, Faculty of Ayurveda, Institute of Medical Sciences, Banaras Hindu University, Varanasi, IND

**Keywords:** eleusine coracana, finger millet, high-resolution mass spectrometry analysis, immunomodulatory activity, ragi millet

## Abstract

Finger millet/ragi (*Eleusine coracana*) is recognized for its nutritional benefits and health-promoting properties, but detailed molecular insights into its bioactive components remain limited. This study employs high-resolution mass spectroscopy (HRMS) to profile the complex biochemical constituents of finger millet, focusing on identifying compounds with potential immunomodulatory effects. Through comprehensive analysis of finger millet extracts, we identified a range of bioactive molecules belonging to different classes of drugs. The data reveal five compounds with significant immunomodulatory activity, suggesting that finger millet may enhance immune system regulation and offer therapeutic potential for immune-related conditions. This research not only deepens our understanding of the molecular makeup of finger millet but also highlights its potential as a functional food with beneficial effects on immune health.

## Introduction

The word "immunomodulation" refers to a variety of potentially therapeutic approaches that elicit a particular immunological response. Essentially, there are two kinds of immunomodulatory responses that can be taken into consideration: one aims to strengthen the immune response when the natural immune response is weak or inadequate, while the other attenuates the natural immunity when it is excessively robust. Therefore, the best immunomodulatory strategy to utilize will depend on the specific condition [1]. Millets have been a core component of the Indian diet for generations, though their cultivation and consumption have declined in recent decades. Today, we are rediscovering their importance in meeting nutritional needs and combating various diseases. This resurgence has earned millets the title 'Shree Anna,' symbolizing their status as the 'mother of all grains'. Rich in nutrients often lacking in modern diets, millet varieties such as ragi, bajra, proso, kodo, jowar, foxtail, little millets, and browntop are excellent sources of essential macronutrients and micronutrients, including iron, zinc, calcium, potassium, protein, and amino acids [2]. Millets are referred to as *Trinadhanya *in Ayurveda. Ayurvedic texts recommend diet-based treatments for various diseases, often highlighting millet as a key component. Finger millet (*Eleusine coracana*) is one of the widely available millets in different parts of India. In the *Kaiyadeva Nighantu* (Ayurvedic treatise), finger millet is referred to with synonyms like *Nartaki *and *Madhulika*. Regarding the pharmacological properties of finger millet as per the Ayurvedic system of medicine, it is described as having a sweet-astringent taste (*Madhura-Kashaya Rasa*), cold potency (*Sheeta Veerya*), and a sweet post-digestive effect (*Madhura Vipaka*). Additionally, it is characterized by lightness (*Laghuta*) and dryness (*Rukshata*). Finger millet is known for its tonic (*Balya*), and nourishing (*Brumhana*) qualities, and for balancing all three bioenergetic principles (*Tridoshas*) of the human body system. Finger millet is rich in iron, calcium, and polyphenols. Traditionally, food made from malted finger millet (ragi) was used for weaning among infants as it provides more energy per feed than other commonly used starch [3]. Finger millet is a wonder cereal that has a wide range of nutritional value, which can combat a wide range of diseases. In India, it is known as ragi, mandua, nachani, and so on [4]. Immunomodulation plays a pivotal role in maintaining health and treating various diseases. By enhancing or suppressing immune responses, it helps in fighting infections, managing autoimmune disorders, treating allergies, supporting cancer therapies, etc. Understanding and managing immunomodulation is vital for advancing medical treatments and improving health outcomes. Hence, this study aims to identify immunomodulatory compounds in the finger millet sample and to evaluate its potential as a supplement for modulating the immune system.

## Materials and methods

Procurement of finger millet

Finger millet for analysis by high-resolution mass spectroscopy (HRMS) was sourced from Yureka Traders in Namakkal, Tamil Nadu, India. The sample was then authenticated by the Department of Ayurvedic Pharmacognosy (Dravyaguna), Faculty of Ayurveda, Institute of Medical Sciences, Banaras Hindu University, Varanasi, India, with the accession number DG/24-25/851. Additionally, it was verified by the Department of Botany under voucher specimen number Poa. 2024/02.

Finger millet, methanol (MeOH), distilled water, and Eppendorf tubes (Eppendorf SE, Hamburg, Germany) were used for the study. The HRMS analysis was performed using the Orbitrap Eclipse Tribrid mass spectrometer, developed by Thermo Fischer Scientific, Waltham, MA. For the phytochemical analysis of small molecules, the Dionex UltiMate 3000 RS UHPLC system was employed (Thermo Fischer Scientific) [5].

Method employed for HRMS analysis

The sample preparation for HRMS analysis started with the addition of an individually optimized sample of finger millet (100 mg) with 1.5 ml of solvent (methanol:water; 80:20) and homogenized using an Eppendorf Thermomixer (Eppendorf SE) Cat 750 rpm for 30 min at 25 °C. Then, the sample was centrifuged (3500 rpm/10 min/25 °C). The supernatant was filtered with a 0.22 µ polytetrafluoroethylene (PTFE) syringe filter, and 4 µl of the filtrate was used as injection volume on the C18 reverse-phase high-performance liquid chromatography (RP-HPLC) column (Hypersil GOLD™, Thermo Fischer Scientific: particle size 1.9 µ, 2.1 mm × 100 mm).

The reversed-phase chromatographic separation starts with a high aqueous phase (+0.1% formic acid) and ends on a highly organic phase (MeOH+ 0.1% formic acid), typically 100% aqueous to 100% organic. The liquid chromatography gradient parameters were as follows: 0 to six minutes 5% MeOH, six to 10 minutes 30% MeOH, 10-20 minutes 50% MeOH, 20-25 minutes 90% MeOH, 25-27 minutes 90% MeOH, and 27-30 minutes 5% with a flow rate of 300 l/min and column oven temperature 40 °C.

The optimized sample of finger millet was tested for metabolomics analysis. Thermo Fisher Scientific high-resolution accurate mass spectrometry system of the model Orbitrap Eclipse Tribrid mass spectrometer coupled with nano liquid chromatography and ultra high-pressure liquid chromatography (Dionex Ultimate 3000 rapid separation liquid chromatography (RSLC)) system, a heated electrospray ionization (HESI) source was used to feed the sample to the mass spectrometer post chromatographic separation. The Orbitrap analyzer was utilized at 60,000 resolutions separately for positive/negative polarity with a mass range (m/z) of 100-1,000, a 35% RF lens, and a 25% normalized automatic gain control (AGC) target, keeping 2.0e5 as the intensity threshold to perform MS-OT (master scan). To obtain ddMS2 OT higher collisional dissociation (HCD), the selection parameters were quadrupole isolation mode with 1.5 isolation window (m/z) HCD activation type, 30, 45, and 60 HCD collision energy (%), 15,000 Orbitrap resolution, and a 20% normalized AGC target.

The raw data obtained from the mass analyzer were performed through the default parameters of Compound Discoverer 3.3.2.31 (Thermo Fischer Scientific) using online databases. The chosen workflow was the Natural Product Unknown ID with both online and local database searches, focused on untargeted food research without statistical analysis. This approach detects and identifies unknown compounds by performing retention time alignment, detecting unknown compounds, and grouping them across all samples. It predicts the elemental compositions for all compounds and filters out chemical backgrounds using blank samples. Compound identification is carried out through mzCloud (HighChem LLC, Bratislava, Slovakia) (ddMS2 and/or DIA), ChemSpider (Royal Society of Chemistry, Cambridge, UK) (using exact mass or formula), and local database searches against mass lists (with or without retention time). Spectral similarity searches are conducted on mzCloud for ddMS2 compounds and spectral distance scoring is applied for ChemSpider and mass list matches [6].

## Results

**Figure 1:**
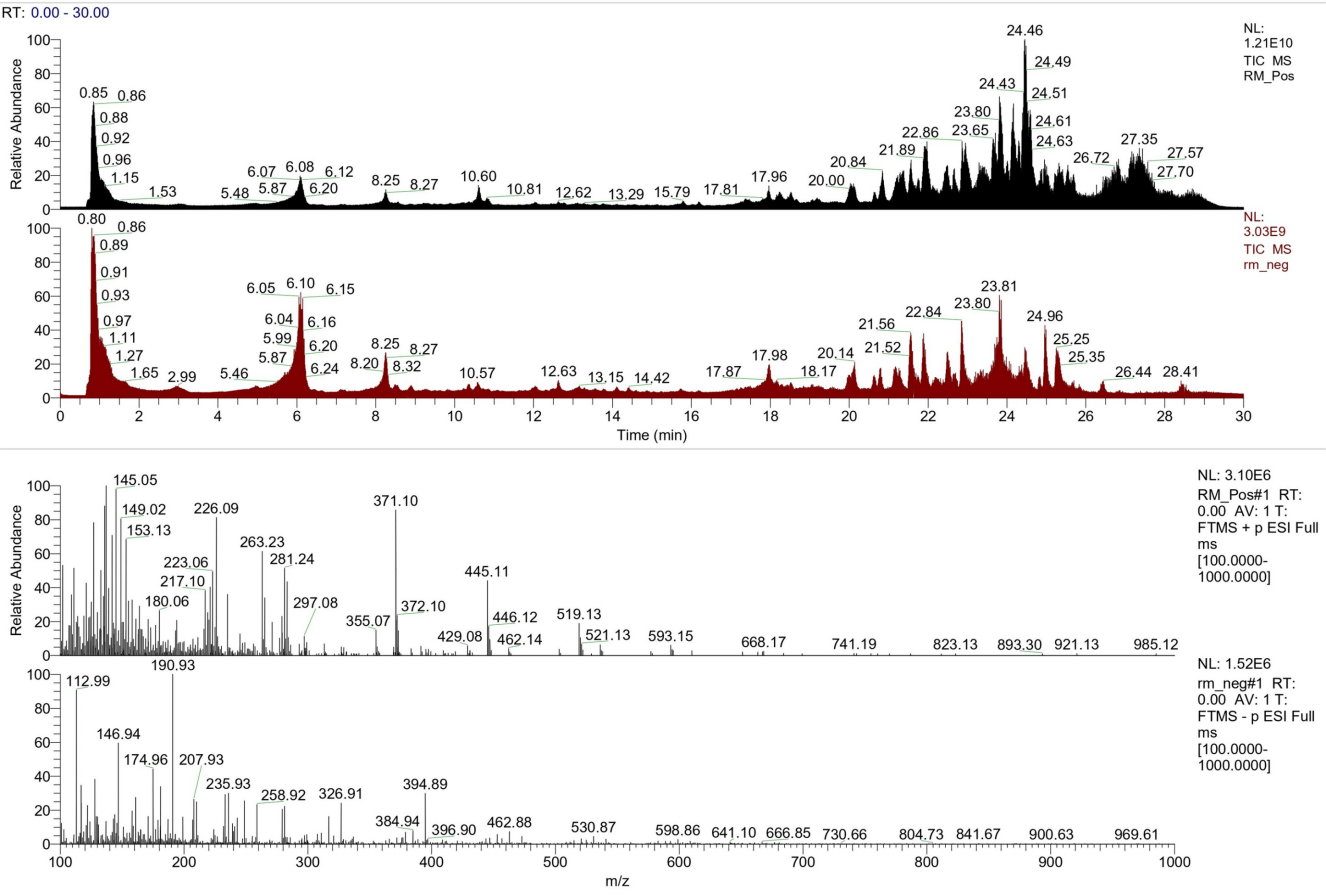
Total ion chromatogram of finger millet obtained by UHPLC-HRAMS analysis of the Eleusine coracana (finger millet/ragi) sample in positive and negative ion modes UHPLC-HRAMS: ultra-high-performance liquid chromatography-high resolution mass spectrometry

The total ion chromatogram (TIC) depicts the total intensity of all ions detected as a function of time. It is a vital tool for interpreting the composition of a sample and understanding the various compounds present in it. It is a visual representation of all the ions detected during a chromatographic separation, allowing us to gain insights into the composition of a sample. The TIC of the components present in finger millet is depicted in Figure 1.

A standard ion chromatogram provides a reference for the identification and quantification of ions in the testing sample. The identified immunomodulatory components/ions in finger millet were quantified and characterized based on their retention times and peak intensities.

Immunomodulatory activities of the phytochemical constituents found in finger millet

By performing HRMS analysis, a total of 1,074 phytochemical constituents were identified in the finger millet sample. Among those phytochemical components, the following components, namely fosfomycin monophosphate, gumperimus, verapamil, leupeptin, and neomycin, exhibit immunomodulatory activity as per the following references.

Fosfomycin Monophosphate

**Figure 2:**
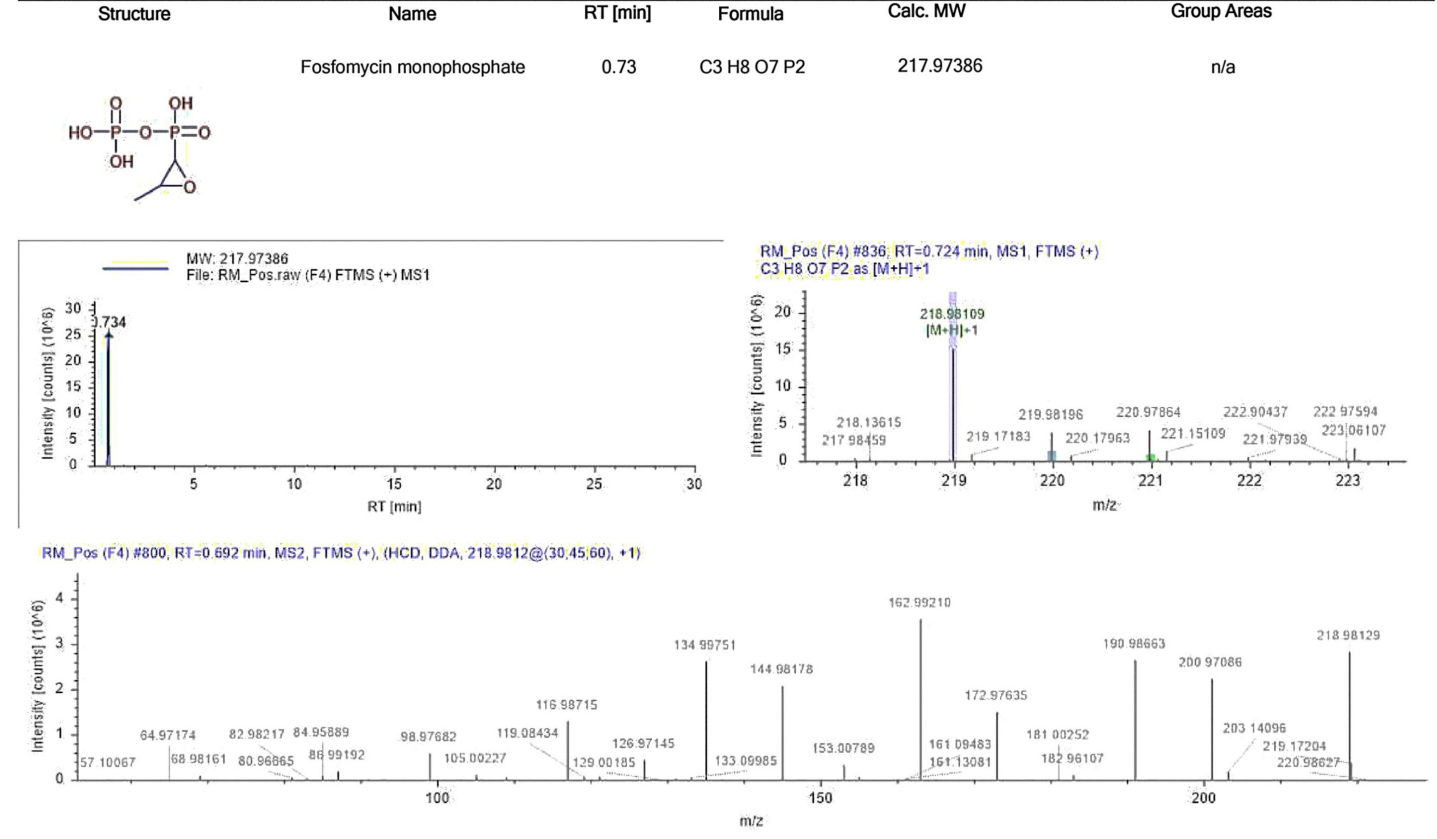
Standard ion chromatogram of fosfomycin monophosphate

Fosfomycin has immunomodulatory effects by influencing the function of lymphocytes, monocytes, and neutrophils. It alters the acute inflammatory cytokine response both in vitro and in vivo. Specifically, fosfomycin reduces the production of tumor necrosis factor-alpha (TNF-α) and interleukin-1 (IL-1) while increasing the production of IL-10, though results regarding IL-6 are contradictory. Additionally, fosfomycin decreases IL-2 production by T cells, inhibits leukotriene B4 (LTB4) production by neutrophils, and lowers IL-8 mRNA expression in monocytes induced by LTB4. It also affects B-cell activation and enhances the ability of neutrophils to kill pathogens, including in patients undergoing chronic hemodialysis or renal transplantation. Compared to other antimicrobials, fosfomycin improves the bactericidal function of neutrophils [7]. The standard ion chromatogram of fosfomycin monophosphate is depicted in Figure 2.

Gusperimus

**Figure 3:**
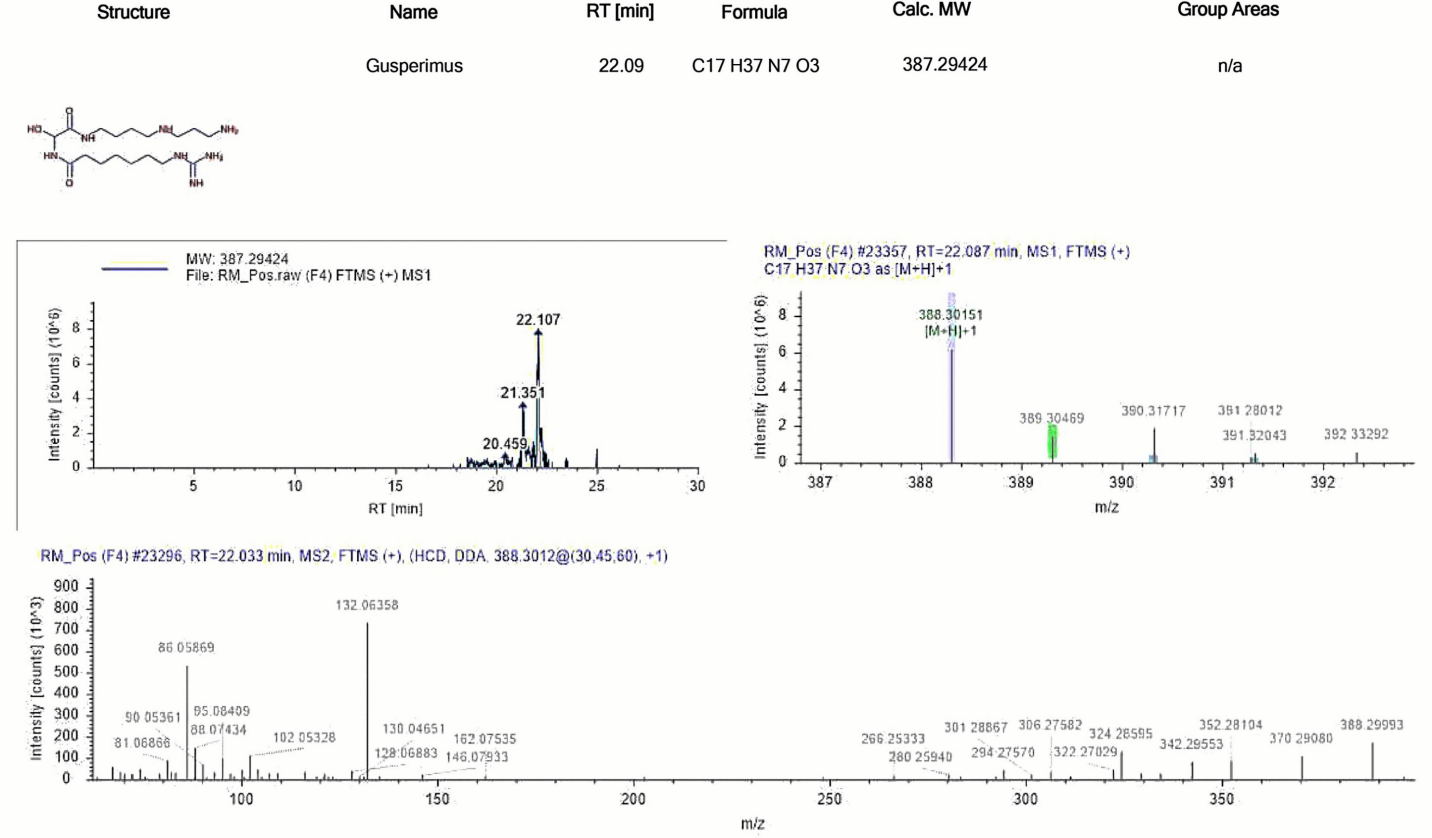
Standard ion chromatogram of gusperimus

Gusperimus (15-deoxyspergualin, Spanidin) is a synthetic derivative of spergualin, a compound originally derived from the soil bacterium *Bacillus laterosporus *and explored for its anti-tumor effects. While it was first studied as a chemotherapy drug, it was later found to have strong immunosuppressive effects. The exact mechanisms behind these effects are complex and not completely understood, as gusperimus influences various aspects of the immune system through multiple processes. It binds to specific regulatory sites on the heat shock proteins Hsc70 and Hsp90, which are believed to inhibit the nuclear translocation of the NF-kB transcription factor. This, in turn, reduces the proliferation and activation of T cells, B cells, monocytes, and dendritic cells and impacts antigen presentation. Gusperimus also inhibits Akt kinase, a crucial signaling molecule involved in cellular survival, cell cycle regulation, and metabolism. Additionally, it interferes with protein synthesis through at least three distinct mechanisms: by down-regulating Akt and thus reducing p70 S6 kinase activity, by binding to Hsc70 and inhibiting the activation of eukaryotic initiation factor 2α (eIF2α), and by directly inhibiting deoxyhypusine synthase, which is essential for activating eukaryotic initiation factor 5A (eIF5A), a protein involved in translation elongation [8]. Gusperimus has been shown to have inhibitory effects on macrophage function and downregulate the production of TNF-α and IL-10 [9]. Gusperimus’s unique mode of action sets it apart from other immunosuppressants. It is approved in Japan for treating steroid-resistant transplant rejection and has shown potential in non-randomized clinical trials for several autoimmune diseases, particularly adeno-associated virus (AAV) and lupus nephritis [8]. The standard ion chromatogram of gusperimus is depicted in Figure 3.

Verapamil

**Figure 4:**
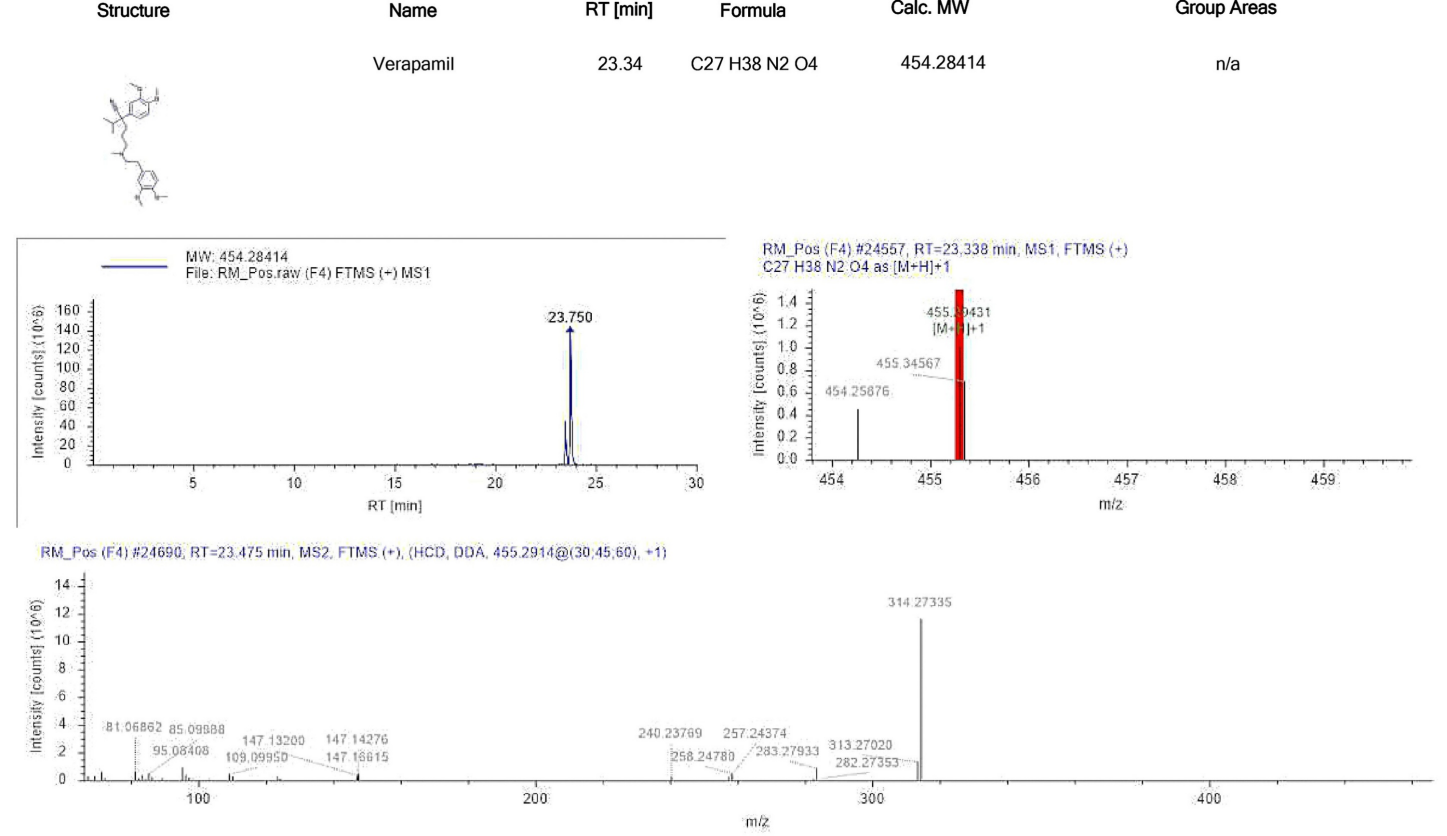
Standard ion chromatogram of verapamil

Verapamil, a calcium channel blocker (CCB) frequently used for treating cardiovascular conditions, works by inhibiting the influx of Ca2+ through L-type calcium channels, which helps relax cardiac and vascular smooth muscle cells. However, CCBs are known for their broad range of effects due to their ability to block various ion channels. Specifically, verapamil has been shown to modulate immune responses in human T cells. It inhibits Ca2+ entry, ATP production, CD25 expression, the buildup of inositol phosphates, cell cycle progression, and the uptake of precursor molecules needed for protein, RNA, and DNA synthesis. Additionally, verapamil affects the generation and function of cytotoxic T cells, as well as disrupting T cell cytoskeleton remodeling, chemotaxis, motility, and transmigration [10]. The RNA sequencing revealed that verapamil influences the thioredoxin system, leading to a gene expression profile characterized by anti-oxidative, anti-apoptotic, and immunomodulatory effects in human islets. These protective changes may collectively account for the overall beneficial effects of verapamil [11]. The standard ion chromatogram f verapamil is depicted in Figure 4.

Leupeptin

**Figure 5:**
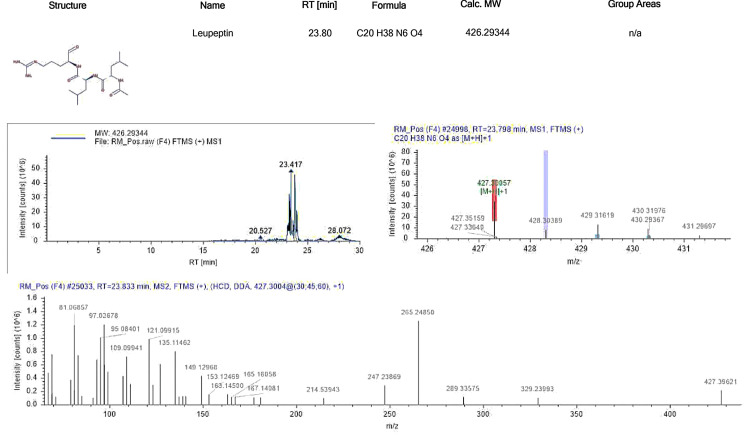
Standard ion chromatogram of leupeptin

Leupeptin, also known as N-acetyl-L-leucyl-L-leucyl-L-argininal, is a peptide metabolite derived from plant-associated actinomycetes with notable public health benefits. It has been shown to counteract increases in TNF-α, IL-1β, IL-6, interferon-gamma (IFN-γ), IL-10, IL-17, IL-5, and IL-15, as well as the pro-inflammatory/anti-inflammatory cytokine ratio, endopeptidases, and Cox-2 levels. Leupeptin's ability to modulate persistent inflammation is attributed to its inhibition of NO-ROS phagocytosis membrane potential, along with its effects on autophagic responses and related inflammatory pathways involving Th1/Th2 and anti-inflammatory cytokines. These findings suggest that leupeptin could serve as an effective alternative anti-inflammatory treatment and highlight its potential as a natural compound for regulating immune responses in inflammatory diseases [12]. The standard ion chromatogram of leupeptin is depicted in Figure 5.

Neomycin

**Figure 6:**
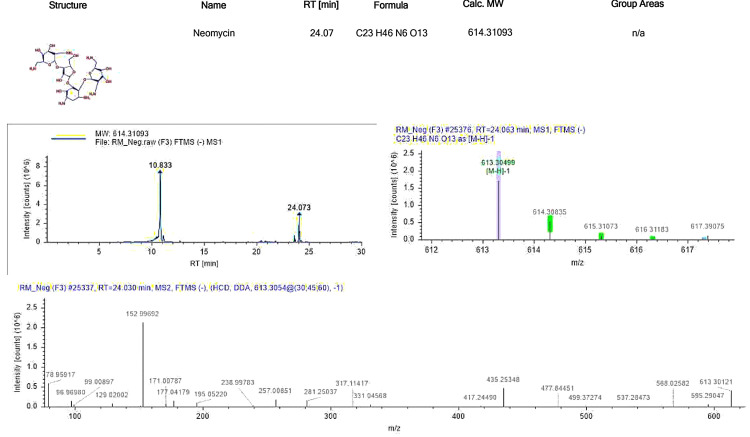
Standard ion chromatogram of neomycin

Neomycin is a polycationic compound that specifically binds to the inositol phospholipids PIP2 and PIP. Its effects vary depending on the concentration, with both stimulatory and inhibitory outcomes reported. Neomycin influences guanine triphosphatase (GTPase) activity, binds to InsP3, and selectively affects thrombin-stimulated platelets. However, there is limited information on how neomycin impacts the signaling pathways in neutrophils. Generally, neomycin is known to inhibit phospholipase C due to its binding to polyphosphoinositides. Despite its inhibitory effects at high concentrations, neomycin at lower concentrations has been found to stimulate NaF-induced leukotriene production, GTPase activity, and actin polymerization [13]. The standard ion chromatogram of neomycin is depicted in Figure 6.

## Discussion

Ayurveda, an ancient system of medicine from India, emphasizes holistic health and balance through various means, including diet. Immunomodulation in Ayurveda involves using medications as well as specific dietary practices to support and regulate the immune system. This approach not only aims to enhance immunity but also to maintain overall well-being and prevent disease. Hence, an initiative has been carried out to highlight the immunomodulatory potential of finger millet by conducting HRMS analysis to identify and isolate the active compounds that may contribute to its immunomodulatory effects. The optimized finger millet sample was subjected to metabolomics analysis using HRMS. The raw data from the mass spectrometer were analyzed with default parameters in Compound Discoverer 3.3.2.31, utilizing online databases for comprehensive evaluation. The HRMS analysis revealed 1,074 phytochemical constituents in the finger millet sample. Notably, five of these compounds, i.e., fosfomycin monophosphate, gusperimus, verapamil, leupeptin, and neomycin, are reported to demonstrate immunomodulatory activity.

Fosfomycin modulates the immune system by reducing IL-2 production in T cells, inhibiting LTB4 production in neutrophils, and decreasing IL-8 mRNA expression in monocytes triggered by LTB4 [7]. Gusperimus demonstrates strong immunosuppressive effects by binding to the regulatory C-terminal regions of heat shock proteins Hsc70 and Hsp90. This binding inhibits the activation of T cells, B cells, monocytes, and dendritic cells. Additionally, gusperimus suppresses Akt kinase activity and protein synthesis [8]. Verapamil affects the immune system by blocking Ca2+ entry, ATP production, CD25 expression, inositol phosphate accumulation, cell cycle progression, and the uptake of molecules needed for protein, RNA, and DNA synthesis [10]. It may also influence immune modulation through the regulation of the thioredoxin system [11]. Leupeptin’s immunomodulatory effects arise from its inhibition of NO-ROS-phagocytosis-membrane potential and its ability to modulate autophagic responses [12]. Neomycin shows concentration-dependent immunomodulatory effects. At high concentrations, it inhibits immune responses by affecting GTPase activity and binding to InsP3. At low concentrations, Neomycin stimulates immune activity by influencing NaF-induced leukotriene production, GTPase activity, and actin polymerization [13].

**Figure 7:**
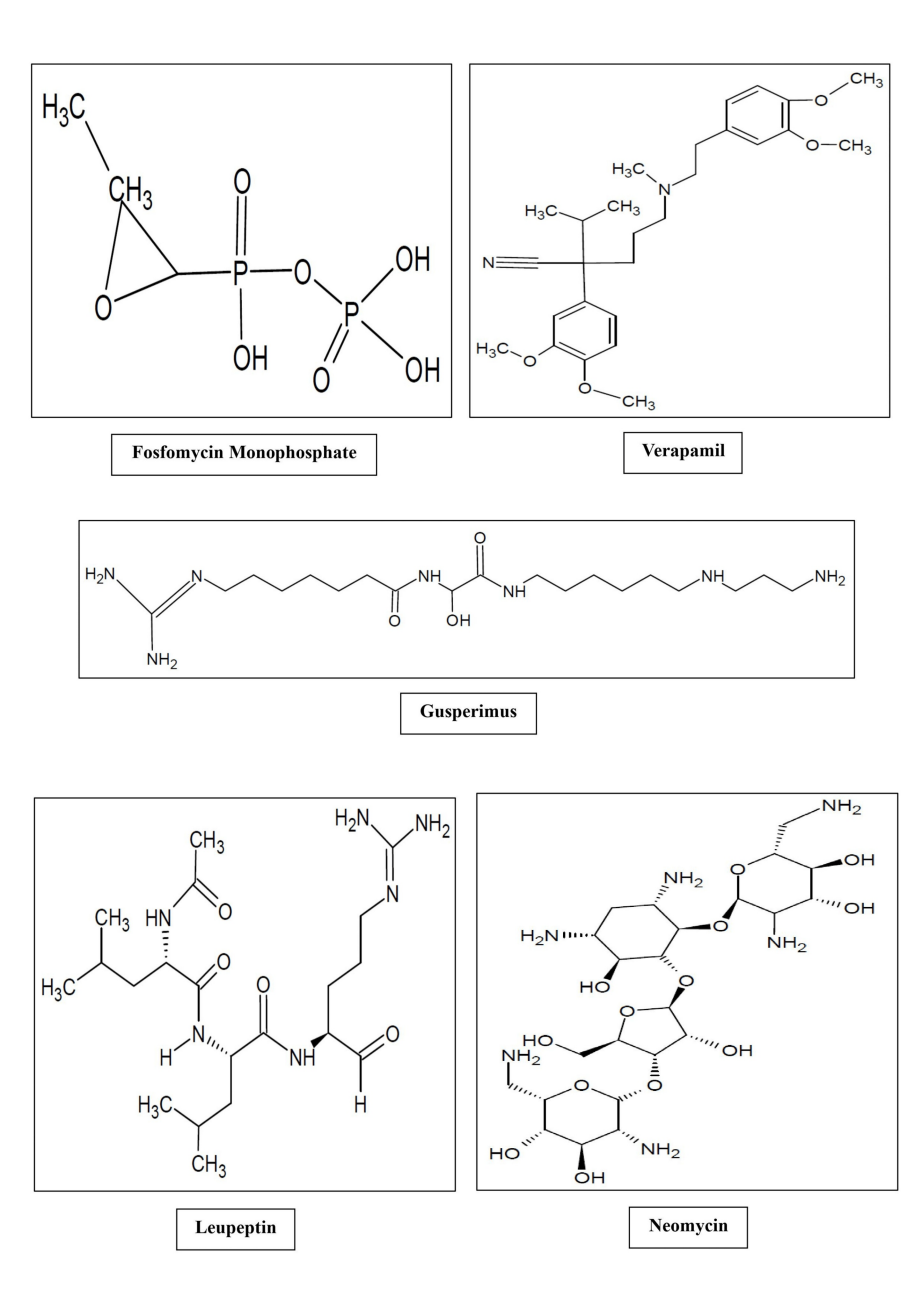
Molecular structure of isolated components from finger millet possessing immunomodulatory potential as identified through high-resolution mass spectrometry analysis This image has been created by the authors.

It can be inferred that the immunomodulatory activity of the specified components is likely associated with their molecular structure, as it profoundly influences their chemical and immunological activities. Factors such as functional groups, shape, stereochemistry, charge distribution, molecular weight, conformational flexibility, and hydrophobic/hydrophilic characteristics all play integral roles in determining how a molecule interacts with biological systems. Hence, the molecular structures of the active components found in finger millet are depicted in Figure 7.

Despite the promising findings regarding the immunomodulatory potential of finger millet, several limitations should be acknowledged. First, the study primarily focused on in vitro analyses, which may not fully replicate the complexities of in vivo interactions and responses within a living organism. Additionally, while five immunomodulatory compounds were isolated, the study did not account for potential interactions with other bioactive compounds present in finger millet that could influence the observed effects. The variability in the immune response based on individual biological factors, such as genetic predisposition and health status, was not considered, which may limit the generalizability of the results. Furthermore, the mechanisms through which these compounds exert their immunomodulatory effects require more extensive investigation, as the current study provides only preliminary insights. These limitations suggest that further research is necessary to validate the findings and explore the full therapeutic potential of finger millet in nutritional immunology.

## Conclusions

Five immunomodulatory compounds from different classes have been isolated. The observed immunomodulatory activity of finger millet is likely due to the synergistic effects of these compounds. In conclusion, the findings from this study, along with existing research, highlight the immunomodulatory potential of finger millet. The differences in their effects on immune stimulation or inhibition suggest that they may exert their actions through various mechanisms. The findings pave the way for further exploration of finger millet's role in nutritional immunology and its application in developing novel immunomodulatory interventions.
